# Surgical ventricular reconstruction and intraoperative cryoablation in a patient with drug-refractory ventricular tachycardia and left ventricular thrombus: a case report

**DOI:** 10.1093/ehjcr/ytae113

**Published:** 2024-03-04

**Authors:** Maryuri Delgado Lopez, Julia Vogler, Anas Aboud, Christian-Hendrik Heeger, Roland Richard Tilz

**Affiliations:** Department of Rhythmology, University Heart Center Lübeck, University Hospital Schleswig-Holstein (UKSH), Campus Lübeck, Ratzeburger Allee 160, Lübeck 23538, Germany; Department of Rhythmology, University Heart Center Lübeck, University Hospital Schleswig-Holstein (UKSH), Campus Lübeck, Ratzeburger Allee 160, Lübeck 23538, Germany; Department of Cardiac and Thoracic Vascular Surgery, University of Schleswig-Holstein, Campus Lübeck, Lübeck, Germany; Department of Rhythmology, University Heart Center Lübeck, University Hospital Schleswig-Holstein (UKSH), Campus Lübeck, Ratzeburger Allee 160, Lübeck 23538, Germany; Department of Rhythmology, University Heart Center Lübeck, University Hospital Schleswig-Holstein (UKSH), Campus Lübeck, Ratzeburger Allee 160, Lübeck 23538, Germany

**Keywords:** Ventricular tachycardia, Left ventricular thrombus, Epicardial ablation, Cryoablation, Surgical ventricular reconstruction, Case report

## Abstract

**Background:**

Despite modern techniques for ablation of ventricular tachycardia (VT), the procedure faces challenges such as deep intramural substrates or inaccessibility of the pericardial space. We aim to present a case of successful surgical treatment of a patient with drug-refractory VT, an apical aneurysm, large left ventricular (LV) thrombus, and recurrent implantable cardioverter defibrillator (ICD) shocks following failed epicardial catheter ablation.

**Case summary:**

A 67-year-old male with a history of ischaemic cardiomyopathy was brought to the emergency room after a syncope because of VT. The VT was terminated by an external cardioversion prior to admission. The ICD interrogation showed an episode of sustained monomorphic VT with eight appropriate but mostly ineffective ICD shocks. An echocardiogram revealed an apical aneurysm with a thrombus. Anticoagulation and antiarrhythmic drug therapy were initiated. Days later, the patient suffered recurrent episodes of sustained VTs, refractory to pharmacological therapy, and epicardial ablation; therefore, following aneurysmectomy and thrombus removal, a reconstruction of the LV and surgical endocardial cryoablation were performed. In addition, ICD extraction was done due to recurrent bacteraemia with *Staphylococcus aureus*. A subcutaneous ICD was later implanted. After surgery, the patient remained free of any VT episodes during 44 months of follow-up.

**Conclusion:**

Combined surgical ventricular reconstruction and intraoperative cryoablation may be considered as an alternative, highly effective therapy in patients with drug-refractory VTs in the setting of a LV thrombus.

**Learning points**

- The safety and efficacy of endocardial ablation in patients with ventricular tachycardia (VT) and the presence of left ventricular (LV) thrombi are still under debate; therefore, the optimal approach after a failed medical therapy remains an individually based decision.
- In patients with drug-refractory VTs in the setting of a LV thrombus when endocardial and/or epicardial ablation fails or is not an option, combined surgical ventricular reconstruction and intraoperative cryoablation should be considered.

## Introduction

Catheter ablation is highly effective for the treatment of refractory ventricular tachycardia (VT), especially in patients with concomitant coronary artery disease, in whom it has shown to result in fewer episodes of recurrence and to reduce the burden of implantable cardioverter defibrillator (ICD) shocks, when compared with escalated antiarrhythmic drug (AAD) therapy.^1,2^ Despite modern ablation techniques, the procedure still faces challenges such as deep intramural substrates or anatomical obstacles such as inaccessibility of the pericardial space due to adhesions or intracavitary thrombi in the left ventricle (LV). The latter is challenging as the treating physician has to weigh the risk of high mortality from drug-refractory VT against thromboembolic events related to VT ablation.^3^ Here, we are presenting a case of successful surgical treatment of a patient with an apical aneurysm, a large LV thrombus, and recurrent ICD shocks following epicardial catheter ablation and failed AAD therapy.

## Summary figure

**Figure:**
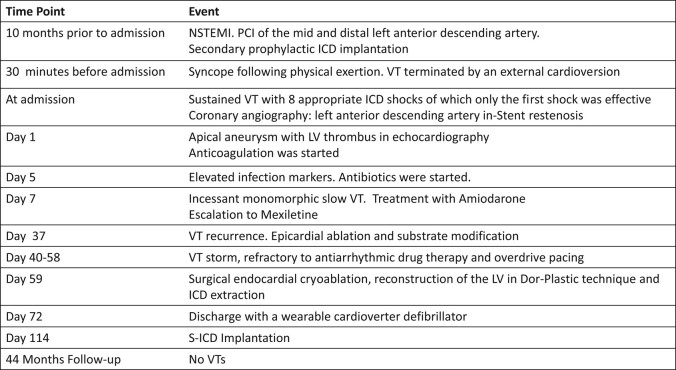


## Case summary

A 67-year-old Caucasian male with a history of ischaemic heart disease with prior percutaneous coronary intervention of the mid and distal left anterior descending artery (LAD) and heart failure with mid-range ejection fraction who underwent ICD implantation 1 year ago for secondary prevention due to recurrent symptomatic VT was brought to the emergency room after a syncope following physical exertion. The physical examination did not reveal any abnormal findings, especially breath and heart sounds were normal on auscultation. The patient was stable, but an ICD interrogation showed an episode of sustained monomorphic VT with eight appropriate ICD shocks of which only the first shock was effective. The VT was terminated by an external direct current (DC) cardioversion by the emergency physician prior to admission.

**Figure 1:**
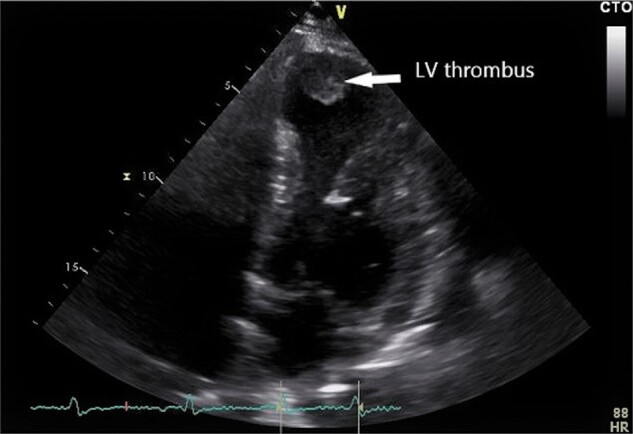
Transthoracic echocardiography. Four-chamber view showing the thrombus in the left ventricle.

A coronary angiogram showed low-grade stenosis of the proximal right coronary artery and a high-grade restenosis of a drug-eluting stent in the medial LAD, which was treated by drug-eluting balloon with good angiographic results. Transthoracic echocardiography revealed cardiac chambers with normal dimensions with a LV ejection fraction of 35%, without relevant valvular diseases. Furthermore, an apical aneurysm with a thrombus of approximately 2 × 2 cm (*Figure 1*) was detected; therefore, an oral anticoagulation therapy was initiated with phenprocoumon as well as an AAD therapy by amiodarone (600–1000 mg/day). With evidence of acute ischaemia and a new LV thrombus, an endocardial catheter ablation was not considered.

On the fifth day of hospitalization, an elevation of leucocytes and laboratory markers for infection was evidenced (*Table 1*); thus, a therapy with rifampicin and later with piperacillin–tazobactam was initiated. Seven days after the admission, the patient developed incessant monomorphic slow VT (125 b.p.m.), which led to haemodynamic instability and was refractory to the treatment with intravenous amiodarone. Despite the escalation of drug therapy using mexiletine (600 mg/day), the patient suffered from recurrent haemodynamically unstable VTs episodes. Mexiletine was preferred over lidocaine due to the possibility of oral administration.

**Table 1:** Main laboratory findings on admission and during the hospital stay

Laboratory findings	Reference	On admission	Day 1	Day 5	Day 7
Leucocytes (× 10^9^/L)	3.6- 10.5	12	9.59	11.5	11.59
C-reactive protein (mg/L)	<5	10	8.37	292	119
Natrium (mmol/L)	136–145	138	138	127	138
Procalcitonin (ng/mL)	<0.05	<0.05		6.77	
Potassium (mmol/L)	3.5–4.5	3.81	4.01	3.69	4.44
Calcium (mmol/L)	2.20–2.55	2.35	2.32	2.25	
Troponin (ng/L)	<14	38	138	77	
CK (U/L)	<190	270	776	109	
CK-Mb (U/L)	<25	4	28.4	—	

CK, creatine kinase.

**Figure 2:**
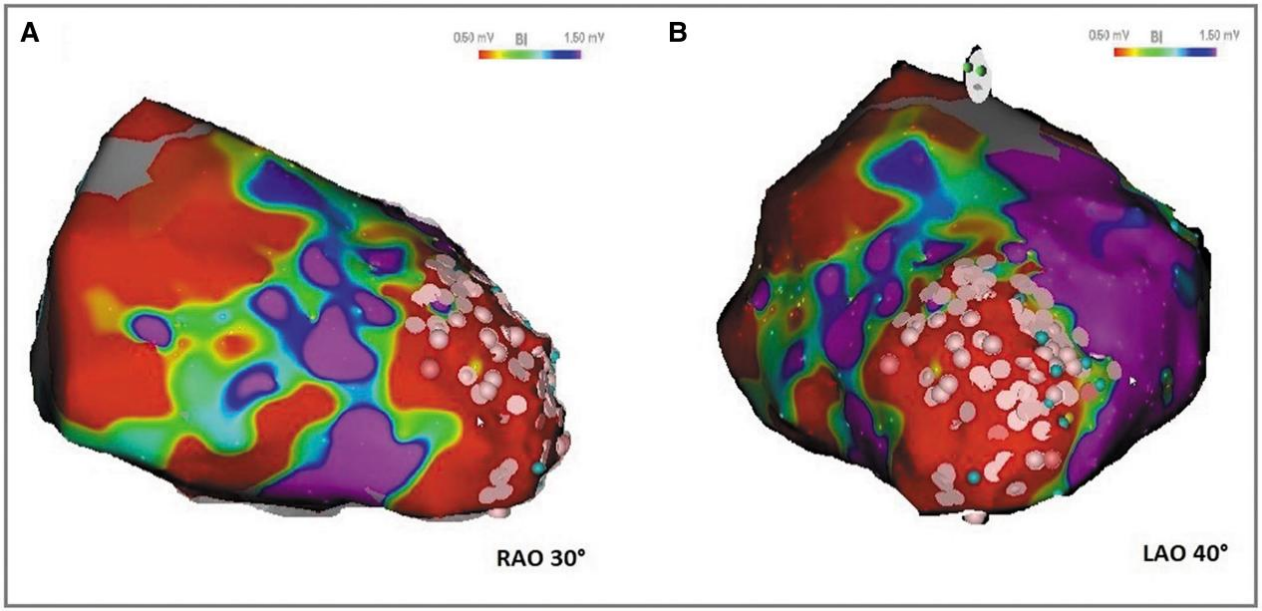
Epicardial bipolar voltage maps. Electroanatomical reconstruction of the left ventricle in right anterior oblique (RAO) 30° (*A*) and left anterior oblique (LAO) 40° (*B*) showing bipolar voltages and areas of ablation. Ablation lesions are displayed as pink dots.

Since the LV thrombus was still present, a purely epicardial ablation was performed. Following dry pericardial puncture, an electroanatomical reconstruction of the epicardial space using a 3D mapping system with superposition of fluoroscopy (CARTO 3, Biosense Webster Inc, Diamond Bar, CA, USA) was performed. To reduce the risk of thromboembolic events, a pure substrate-based approach was chosen using voltage map and pace mapping. The voltage map revealed a VT substrate (low voltage and late potentials) in the apical LV (*Figure 2*). Perfect pacemap of the clinical VT was achieved in that area and substrate modification with the endpoint of elimination of all late potentials was performed using irrigated radiofrequency ablation.

Three days after ablation, the patient developed another VT storm refractory to AAD therapy. Thus, the patient was scheduled for surgical VT therapy. The therapeutic options were extensively discussed with the patient at all times.

**Figure 3:**
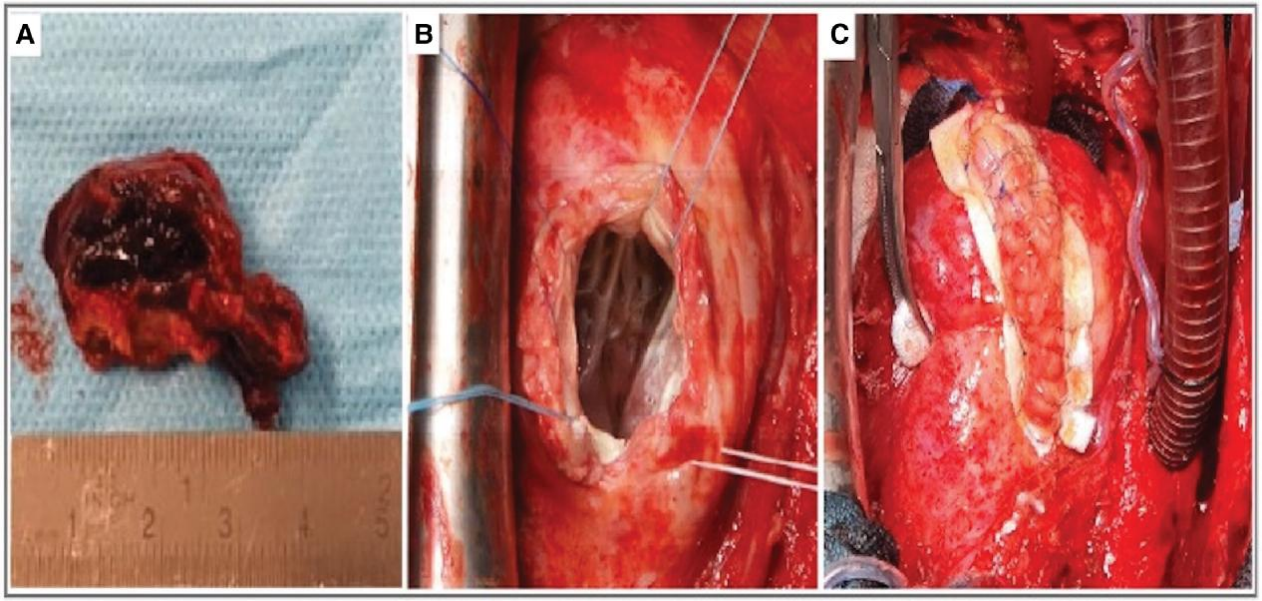
Intraoperative views. Intraoperative views show: (*A*) Thrombus. (*B*) Left ventricular cavity after thrombus resection. (***C***) Left ventricle reconstruction with Dacron patch and purse-string suture.

Twenty-two days after the epicardial ablation, the thrombus was removed, and following aneurysmectomy, the reconstruction of the LV was done in Dor–Plastic technique using a Dacron patch (*Figure 3*). Additionally, a surgical endocardial cryoablation using the ATRICURE cryoICE probe was applied without mapping behind the scar area (‘blind anatomical ablation’). As the patient had a history of recurrent bacteraemia with *Staphylococcus aureus* and the source of infection was not identified, the ICD system was extracted during the same surgery. The procedure itself was uneventful.

The microbiological culture of the removed lead and subsequent blood cultures remained negative. Transthoracic echocardiography after surgery revealed an improved ejection fraction (45%) and no signs of remaining LV thrombus.

The patient was discharged 13 days after surgery with a wearable cardioverter defibrillator. Upon discharge, antiarrhythmic therapy with amiodarone (200 mg/day) and oral anticoagulation with phenprocoumon were prescribed. A subcutaneous ICD was implanted 42 days after discharge. The patient remained free of any VT episodes during 44 months of follow-up.

## Discussion

The management of patients with electrical storm often requires a complex approach, which could include ICD reprogramming, AAD therapy, sedation, catheter ablation, and mechanical circulatory support. In selected patients, autonomic modulation may be considered.^1^ Our case shows successful surgical treatment of incessant monomorphic VT in a patient with ischaemic cardiomyopathy and a large LV thrombus after failed medical therapy and epicardial ablation.

In patients with underlying structural heart disease, sustained monomorphic VTs are typically caused by scar-related re-entry. For its treatment, catheter ablation is increasingly performed.^4^ In the absence of an urgent indication for VT ablation, there is a clear recommendation to start the patient on oral anticoagulation and AAD therapy for a period of time and re-assess the presence of the LV thrombus. Although the presence of a laminated thrombus is not a perceived contraindication according to the guidelines, the feasibility, safety, and efficacy of endocardial ablation in patients with VT and the presence of LV thrombi are still under debate, and the optimal approach after a failed medical therapy remains an individually based decision.^5^

Josephson *et al*.^6^ demonstrated that the thrombus may limit the efficacy of mapping, although no embolic complications occurred. On the other hand, Rao *et al*.^7^ denoted that even when the clinical VT has a critical region within the LV apex, a successful endocardial ablation is still possible with ablation at borderzone regions in patients with organized thrombi. An endovascular two-filter-based cerebral protection system may be considered in highly selected patients undergoing VT ablation in this setting to prevent cerebral embolism as shown in a recent case series.^8^

In front of a clear contraindication for an endocardial access like a huge and possible mobile LV thrombus as seen in our patient, a pure epicardial approach without VT induction may be necessary.^9^ Studies have suggested epicardial ablation as first-line strategy in selected patients since a substantial number of VT circuits involve the subepicardial layer. Notwithstanding, in patients without an intracavitary thrombus obviating an endocardial approach, a real contribution considering the higher risk for procedural complications remains unclear.^10^ In our report, we demonstrate that a pure epicardial VT ablation without VT induction is feasible, but endpoints are difficult to verify since only epicardial late potential elimination can be evaluated. Without VT induction, we were not able to assess non-inducibility of all the clinical VT as endpoint, which might explain VT recurrence in our case.

Our patient developed recurrent VTs after epicardial ablation despite the escalation of AAD therapy; the ICD function could not be assessed due to the LV thrombus. Furthermore, he had recurrent episodes of bacteraemia, suggesting device infection. Since the patient did not present signs of infection at the time of admission, bacteraemia alone could not be the trigger for VT. A surgical approach combining surgical ventricular reconstruction (SVR) and intraoperative cryoablation may be considered as a bailout strategy in cases with drug-refractory VTs, ischaemic cardiomyopathy, and LV remodelling. Therefore, we decided to perform SVR, surgical ablation, and ICD extraction. Eliminating the scar tissue accounting for the electric disturbance by SVR does not only result in a reduction of VTs but may also reduce the rate of hospitalization and improve ventricular function in patients with heart failure caused by coronary artery disease.^11,12^

Although surgical therapy seems to be effective for the treatment of VT post-myocardial infarction, it is associated with significant morbidity and mortality. The most common complications after SVR are bleeding and deterioration of LV function, which can be mitigated by using the patch technique that minimizes the tensile stress on the ventricular wall and thus reduces the risk of rupture and of bleeding.^13^

Transvenous catheter-based electrical isolation of the entire ischaemic substrate is mimicking the surgical approach while being less invasive. Since patients may suffer from recurrent VTs after surgical and transvenous VT ablation, early ICD implantation is recommended.^1^

In patients with refractory VT, the role of stereotactic arrhythmia radioablation (STAR) should be discussed as a potential alternative.^14^ Due to the lack of evidence on the acute effectiveness of STAR in stabilizing patients with recurrent electrical storm in the setting of LV thrombus when the patient presented, this option was not considered.

Recently, novel strategies, such as pulsed field ablation, have been already successfully tested in humans for the treatment of VT.^15^ However, the data are scarce.

## Conclusion

Combined SVR and intraoperative cryoablation may be considered as an alternative, highly effective therapy in patients with drug-refractory VTs in the setting of a LV thrombus when endocardial and/or epicardial ablation fails or is not an option.

## Data Availability

The data underlying this article will be shared on reasonable request to the corresponding author.

## Lead author biography

Maryuri Delgado Lopez is a cardiology resident at Heart Center Lübeck, Germany. She graduated in Medicine in 2017 at the Catholic University of Guayaquil, Ecuador. Her main areas of interest are novel mapping and ablation technologies.
